# A gastric dieulafoy lesion presenting with life-threatening upper gastrointestinal hemorrhage during recovery from hypertriglyceridemic acute pancreatitis: a case report

**DOI:** 10.3389/fmed.2026.1949159

**Published:** 2026-09-29

**Authors:** Nan Liu, Meina Lang, Ning Wang, Run Liu, Yulan Liu, Zhengrong Guo

**Affiliations:** 1 Department of Gastroenterology, Shijiazhuang People’s Hospital, Shijiazhuang, Hebei, China; 2 Department of Gastroenterology, Peking University People’s Hospital, Beijing, China

**Keywords:** case report, continuous renal replacement therapy, dieulafoy lesion, endoscopic hemostasis, hemoclips, hypertriglyceridemic acute pancreatitis, plasma exchange, upper gastrointestinal bleeding

## Abstract

**Background:**

Dieulafoy lesions are an uncommon but potentially life-threatening cause of non-variceal upper gastrointestinal bleeding, caused by rupture of a large-caliber submucosal artery through a minute mucosal defect; diagnosis is difficult because bleeding is intermittent and the surrounding mucosa appears nearly normal. Hemorrhage from a gastric Dieulafoy lesion during recovery from hypertriglyceridemic acute pancreatitis (HTG-AP) has rarely been reported.

**Case presentation:**

A man in his twenties with severe obesity presented with acute epigastric pain after alcohol and a high-fat meal. Serum triglycerides were 53.67 mmol/L (reference 0.56–1.70), and CT confirmed acute pancreatitis. Because of extreme hypertriglyceridemia and evolving organ dysfunction, he received intensive lipid-lowering therapy, plasma exchange, continuous renal replacement therapy, and supportive care in the ICU, and improved. During recovery he developed sudden massive hematemesis with tachycardia, hypotension, and a fall in hemoglobin to 65.9 g/L. Emergency endoscopy showed extensive intragastric clots and could not locate the source. After stabilization, repeat endoscopy revealed an isolated thrombus-like protrusion with active oozing on the lesser curvature near the cardia, with otherwise normal mucosa, consistent with a Dieulafoy lesion; three metallic clips achieved immediate hemostasis. Contrast-enhanced CT excluded pseudoaneurysm, splenic-vein thrombosis, gastric varices, and other pancreatitis-related vascular complications. No recurrence occurred during hospitalization or approximately seven months of follow-up, with healed mucosa on later endoscopy.

**Conclusion:**

A gastric Dieulafoy lesion is a rare but life-threatening cause of bleeding during recovery from HTG-AP. A nondiagnostic first endoscopy because of clot retention does not exclude it, and persistent bleeding warrants repeat endoscopy after stabilization and gastric clearance. Bleeding should not be attributed solely to stress injury or anticoagulation; an underlying structural lesion should be sought. Clipping achieved durable hemostasis in this patient, but a single case cannot establish comparative efficacy.

## 1 Introduction

Acute pancreatitis (AP) is one of the most common gastrointestinal emergencies worldwide and remains associated with substantial morbidity and healthcare utilization. According to the revised Atlanta classification, AP is categorized as mild, moderately severe, or severe based on the presence and duration of organ failure and local or systemic complications (1). Among the various etiologies of AP, hypertriglyceridemic acute pancreatitis (HTG-AP) has emerged as an increasingly recognized subtype, accounting for approximately 2%–10% of all AP cases and a considerably higher proportion in younger patients and those with metabolic disorders (26). The pathogenesis of HTG-AP is closely associated with excessive circulating triglycerides. When triglyceride concentrations become markedly elevated, pancreatic lipase hydrolyzes triglyceride-rich lipoproteins, generating large amounts of free fatty acids (2, 3). These free fatty acids can induce pancreatic acinar cell injury, microvascular dysfunction, endothelial damage, and amplification of the systemic inflammatory response, thereby contributing to a more severe disease course than that observed in many other forms of AP (4). Consequently, severe HTG-AP may require intensive management, including insulin-based lipid-lowering therapy and, in selected patients, plasma exchange or continuous renal replacement therapy (CRRT) for extreme hypertriglyceridemia and organ support.

Gastrointestinal bleeding is a recognized complication in patients with severe or moderately severe AP (5). Common etiologies include stress-related mucosal disease, peptic ulcer disease, erosive gastritis, Mallory–Weiss syndrome, coagulation abnormalities, anticoagulation-associated bleeding, and pancreatitis-related vascular complications such as pseudoaneurysm rupture or gastric varices secondary to splenic vein thrombosis (6). Because many patients with severe pancreatitis receive anticoagulation during extracorporeal therapies and may exhibit coagulation–fibrinolytic disturbances, identifying the exact source of gastrointestinal bleeding can be diagnostically challenging. In contrast, Dieulafoy lesion is an uncommon but potentially life-threatening vascular anomaly characterized by a large-caliber submucosal artery that protrudes through a minute mucosal defect. Although it accounts for only a small proportion of non-variceal upper gastrointestinal bleeding, rupture of the lesion may result in sudden and massive hemorrhage (7–9) Diagnosis is frequently difficult because the surrounding mucosa often appears nearly normal, bleeding may be intermittent, and the responsible vessel can be obscured by retained blood clots during emergency endoscopy (10–14).

To date, reports describing Dieulafoy lesion-associated gastrointestinal bleeding in the setting of acute pancreatitis remain exceedingly rare, and massive gastric Dieulafoy bleeding occurring during the recovery phase of HTG-AP has seldom been documented. We report this case to highlight the diagnostic challenge posed by a gastric Dieulafoy lesion during recovery from HTG-AP, to emphasize that a nondiagnostic initial endoscopy should not preclude repeat examination when evidence of bleeding persists, and to illustrate the role of endoscopic mechanical hemostasis in its management (1, 15). The clinical course, diagnostic reasoning, and treatment of the patient are described below.

## 2 Case presentation

A man in his twenties presented to the emergency department on hospital day 1 with persistent upper abdominal pain of 1 day's duration following alcohol consumption and ingestion of a high-fat meal. He was 187 cm tall and weighed 140 kg, corresponding to a body mass index (BMI) of 40.03 kg/m (2). He had no history of diabetes mellitus, hypertension, hyperlipidemia, pancreatitis, gastrointestinal bleeding, or gallstone disease. There was no known family history of pancreatic disorders or hereditary lipid abnormalities.

On admission, he was tachycardic (heart rate 131 beats/min), tachypneic (respiratory rate 33 breaths/min), and hypertensive (blood pressure 183/106 mmHg), with a temperature of 36.3 °C. Physical examination revealed upper abdominal tenderness without rebound tenderness or guarding. Laboratory investigations demonstrated markedly elevated serum triglycerides of 53.67 mmol/L (reference range 0.56–1.70 mmol/L), total cholesterol 21.81 mmol/L (reference range 2.80–5.18 mmol/L), pancreatic amylase 268.50 U/L (reference range 8–53 U/L), and total amylase 327 U/L (reference range 35–135 U/L). Abdominal computed tomography (CT) findings were consistent with acute pancreatitis. Based on the clinical presentation, laboratory findings, and imaging results, a diagnosis of HTG-AP was established.

Given the extreme hypertriglyceridemia, pronounced systemic inflammatory response, and evolving organ dysfunction, he was admitted to the emergency intensive care unit, where he received bowel rest, aggressive fluid resuscitation, somatostatin, ulinastatin, intravenous insulin-based triglyceride-lowering therapy, fenofibrate, acipimox, and proton pump inhibitor therapy. Plasma exchange and continuous renal replacement therapy (CRRT) were escalated in this selected patient for refractory severe hypertriglyceridemia and evolving organ dysfunction after initial medical therapy; these interventions were individualized to his clinical condition and are not presented as routine treatment for all patients with HTG-AP. Low-molecular-weight heparin was used only during extracorporeal circulation as clinically indicated. Antimicrobial treatment and other supportive care were provided as required. During the first 48 h of hospitalization, the Ranson score increased from 1 to 3, suggesting progression toward moderately severe acute pancreatitis. Following intensive treatment, abdominal pain gradually improved, inflammatory markers decreased, and serum triglyceride levels rapidly declined. Dynamic changes in hemoglobin, triglyceride, and C-reactive protein levels throughout hospitalization are shown in Figure 1, and the major clinical events are summarized in Table 1.

**Figure 1:**
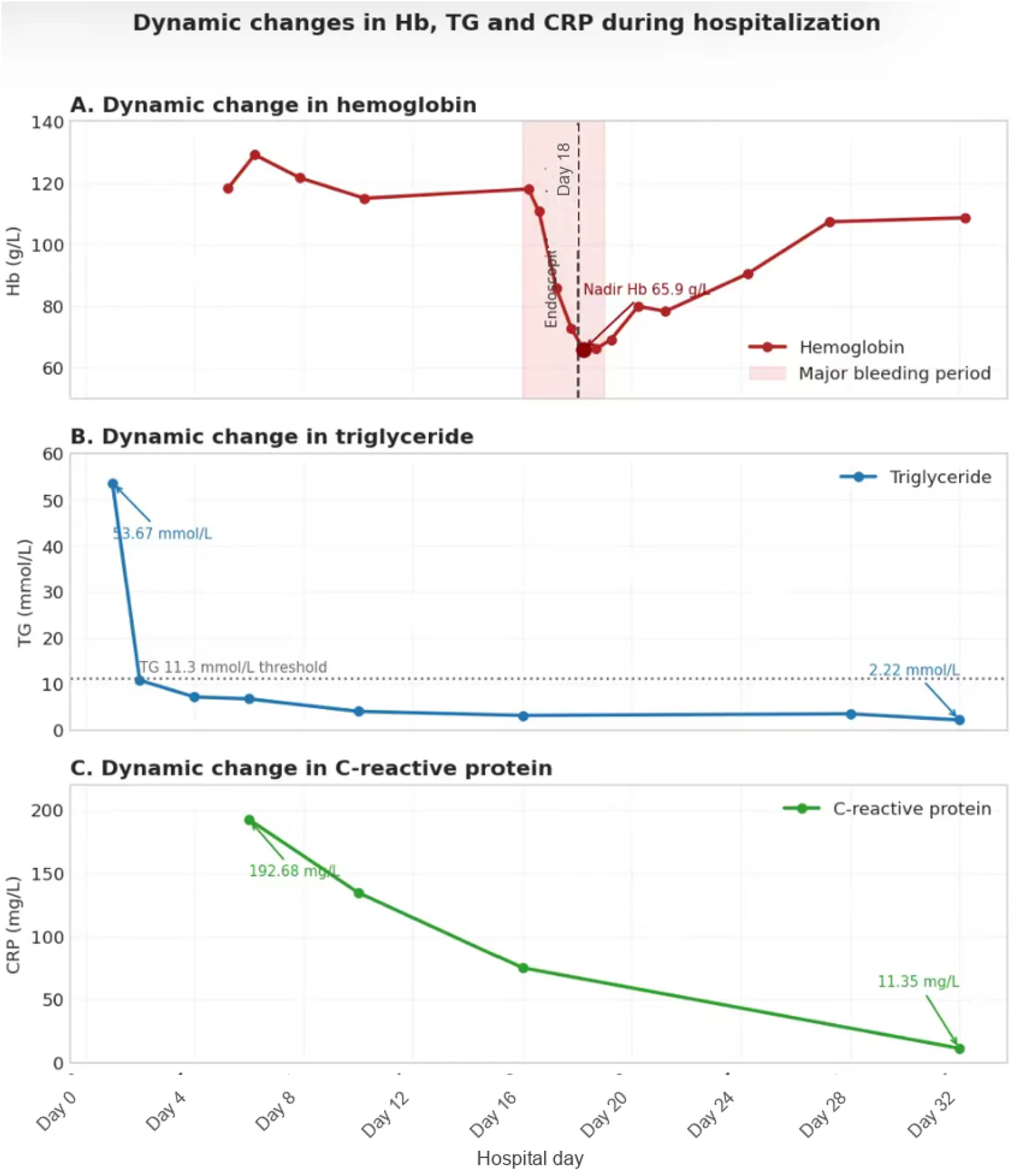
Dynamic changes in hemoglobin **(A)**, triglycerides **(B)**, and C-reactive protein **(C)** during hospitalization, showing the hemorrhage-related fall and recovery of hemoglobin, the rapid reduction of triglycerides from 53.67 mmol/L, and the resolution of inflammation.

**Table 1:** Clinical timeline of the hospitalization and follow-up. Relative clinical time points are used to protect patient anonymity.

Time point	Clinical event
Hospital day 1	Admission with HTG-AP and markedly elevated triglycerides (53.67 mmol/L)
Hospital days 1–5	Intensive lipid-lowering therapy, plasma exchange, CRRT, and organ-supportive care; clinical improvement
Hospital day 5	Transfer from the intensive care unit to the gastroenterology ward; jejunal enteral nutrition initiated
Hospital day 15	Bloody drainage from the gastrointestinal decompression tube; anticoagulation withheld
Hospital day 16	Sudden massive hematemesis with hemodynamic instability; initial endoscopy limited by extensive clot retention; no bleeding source identified
Hospital day 18	Repeat endoscopy after stabilization identified a gastric Dieulafoy lesion near the cardia; three metallic clips applied with complete hemostasis
Approximately 2 weeks after hemostasis	Follow-up endoscopy: clips in place without recurrent bleeding; small superficial gastric angle ulcer biopsied (chronic gastritis; H. pylori negative)
Approximately 7 months after bleeding	Long-term follow-up endoscopy: clips retained, mucosa healed, no recurrent gastrointestinal hemorrhage

On hospital day 5, the patient was transferred to the gastroenterology ward in stable condition. His hemoglobin concentration at transfer was 118.2 g/L (reference range 130–175 g/L). Given the severity of pancreatitis and the prolonged critical illness, jejunal enteral nutrition was initiated to provide enteral feeding while minimizing gastric stimulation and improving nutritional tolerance. However, on hospital day 15, a small amount of bloody fluid was noted in the gastrointestinal decompression tube. Anticoagulation therapy was immediately discontinued, and proton pump inhibitor treatment was intensified. Despite these measures, the patient suddenly developed massive hematemesis on the following day (hospital day 16), accompanied by tachycardia and hypotension. Acute upper gastrointestinal hemorrhage was suspected, and he was transferred back to the intensive care unit for emergency management. Blood transfusion, fluid resuscitation, intravenous proton pump inhibitor therapy, and endotracheal intubation for airway protection were promptly initiated. Emergency esophagogastroduodenoscopy demonstrated extensive blood accumulation within the upper gastrointestinal tract. Large amounts of fresh blood and dark-red clots filled the stomach, particularly the cardia, gastric fundus, and upper gastric body. Despite repeated irrigation and suction, visualization remained severely limited because of extensive clot retention, and no definitive bleeding source could be identified (Figure 2).

**Figure 2:**
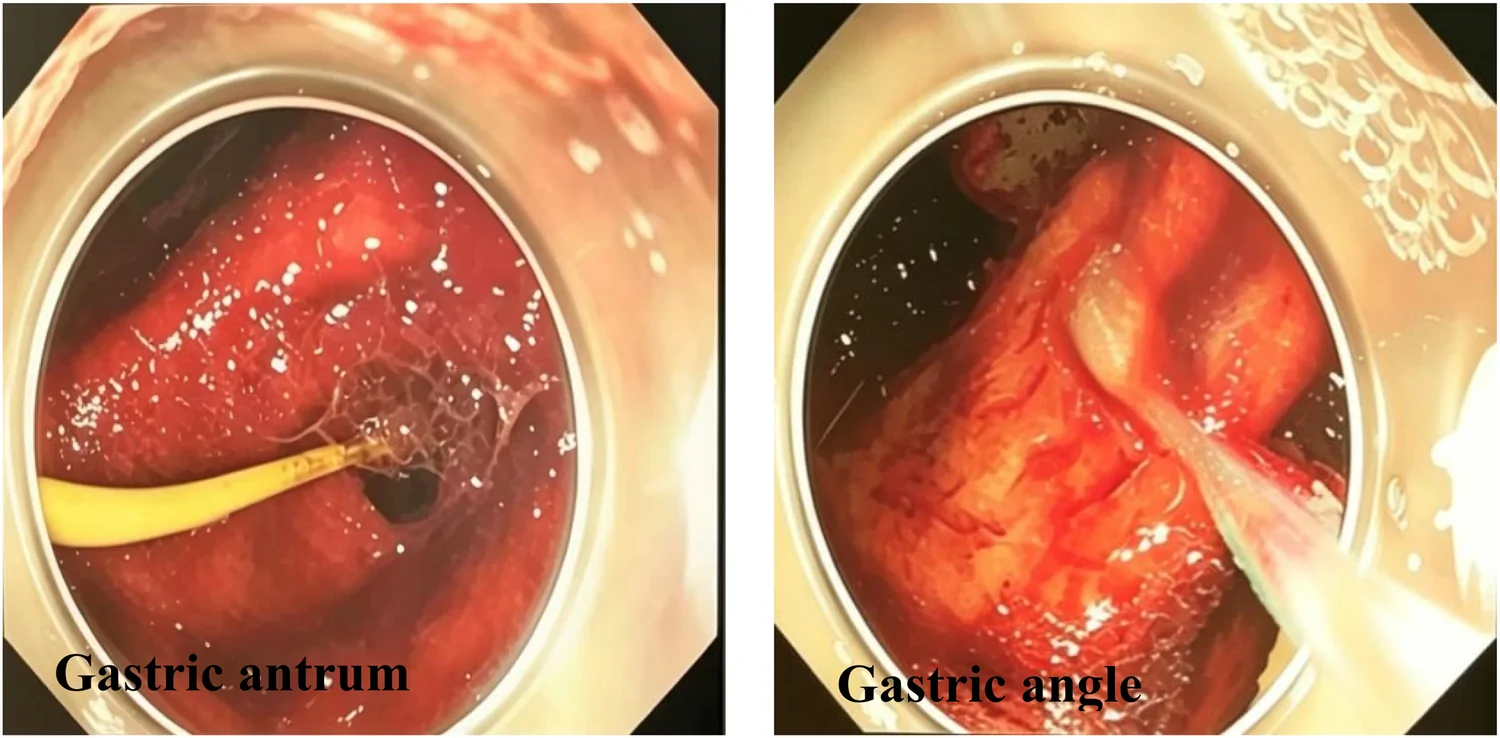
Initial endoscopy on hospital day 16: extensive fresh blood and clots severely limited visualization, and no bleeding source was identified.

Following the initial endoscopy, the patient continued to exhibit evidence of ongoing blood loss. His hemoglobin level progressively declined and reached a nadir of 65.9 g/L (reference range 130–175 g/L) on hospital day 18. Because persistent hemorrhage was suspected, repeat upper gastrointestinal endoscopy was performed after hemodynamic stabilization. During the second examination, repeated irrigation and clot removal provided improved visualization of the proximal stomach. An isolated thrombus-like protrusion was identified on the lesser curvature of the upper gastric body near the cardia. Fresh blood adhered to the lesion surface, and active oozing was observed. Importantly, the surrounding mucosa appeared essentially normal without obvious ulceration or erosion. These findings strongly suggested a gastric Dieulafoy lesion. Three metallic clips were sequentially deployed at the base of the lesion, resulting in immediate and complete hemostasis (Figure 3).

**Figure 3:**
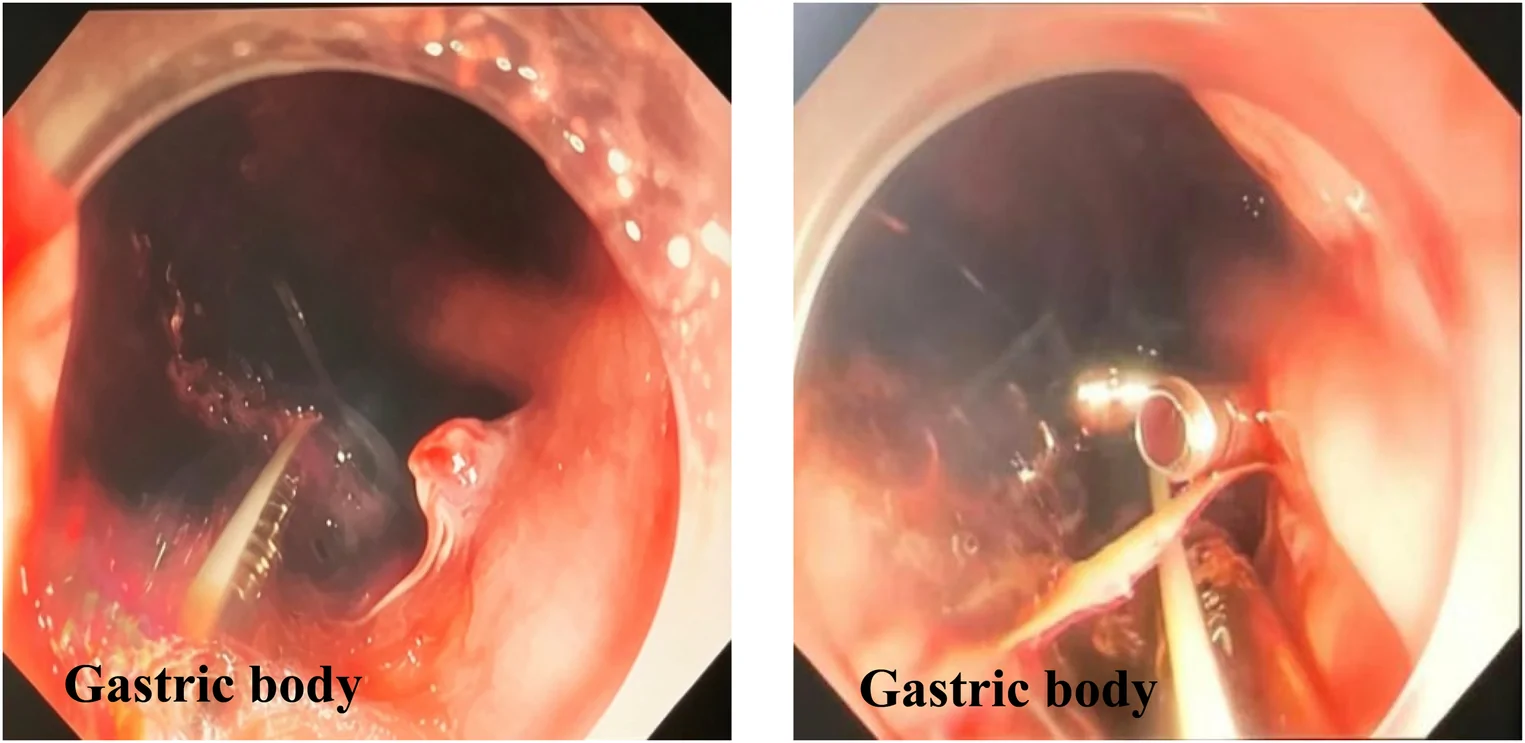
Repeat endoscopy on hospital day 18: an actively oozing dieulafoy lesion near the cardia with otherwise normal mucosa; three metallic clips achieved complete hemostasis.

Following successful endoscopic treatment, the patient received continued blood transfusion, proton pump inhibitor therapy, fluid replacement, and supportive management. No recurrent hematemesis or melena occurred, and his hemodynamic status gradually stabilized. A follow-up gastroscopy performed approximately two weeks after hemostasis demonstrated that all three clips remained firmly attached at the treatment site without evidence of recurrent bleeding. Mild mucosal congestion was present around the clips. In addition, a small superficial ulcer was identified at the gastric angle. Histopathological examination revealed chronic gastritis with mild inflammatory activity and no evidence of malignancy. Helicobacter pylori staining was negative (Figure 4).

**Figure 4:**
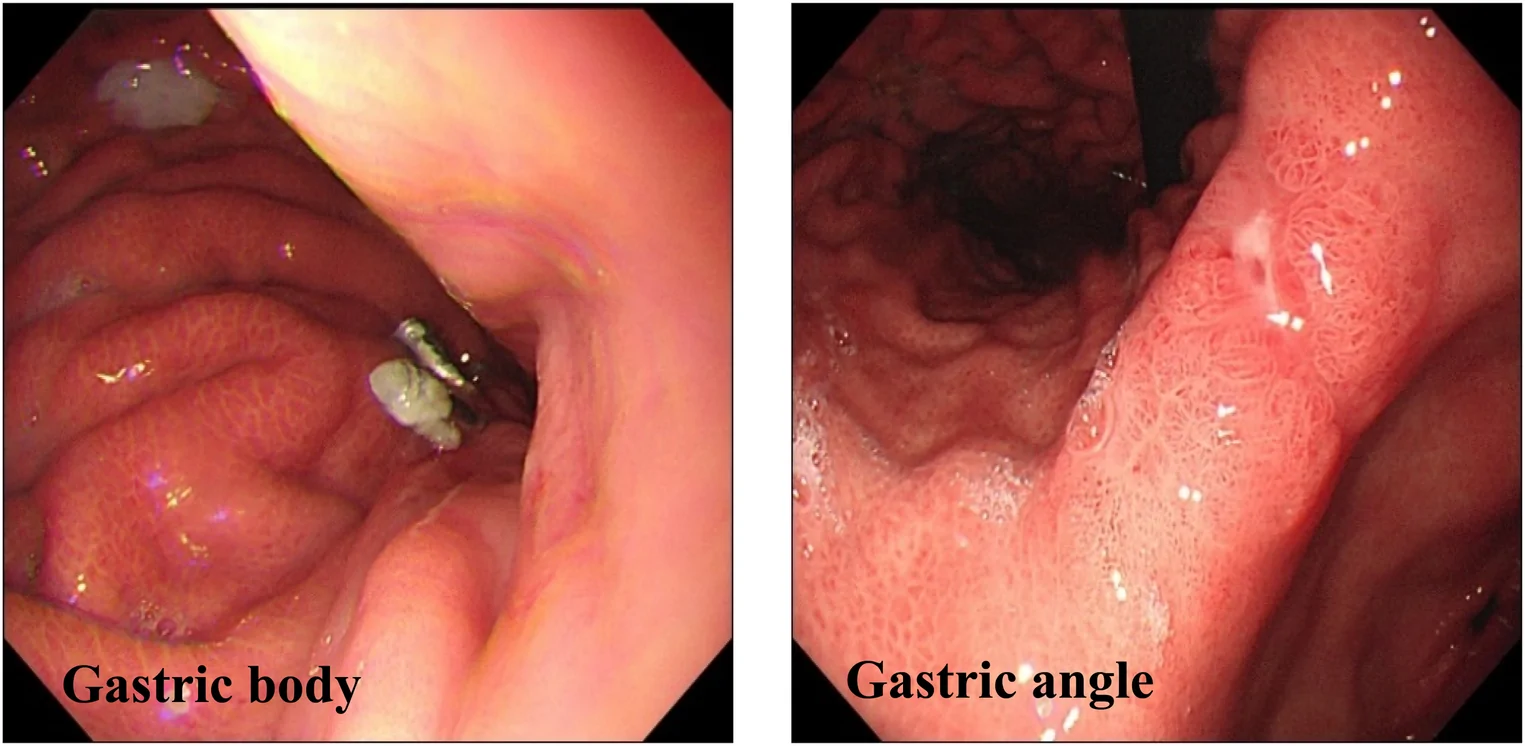
Two-week follow-up: clips in place without rebleeding; the biopsied gastric-angle ulcer showed chronic gastritis with negative H. pylori staining and no malignancy.

To further investigate potential causes of gastrointestinal bleeding, serial contrast-enhanced CT examinations were reviewed. Although extensive peripancreatic inflammatory exudation was present, no pseudoaneurysm, splenic vein thrombosis, gastric varices, or other pancreatitis-related vascular abnormalities were detected (Figure 5). Dynamic coagulation monitoring demonstrated only mild fluctuations in coagulation parameters and did not support severe anticoagulant-related coagulopathy as the primary cause of hemorrhage.

**Figure 5:**
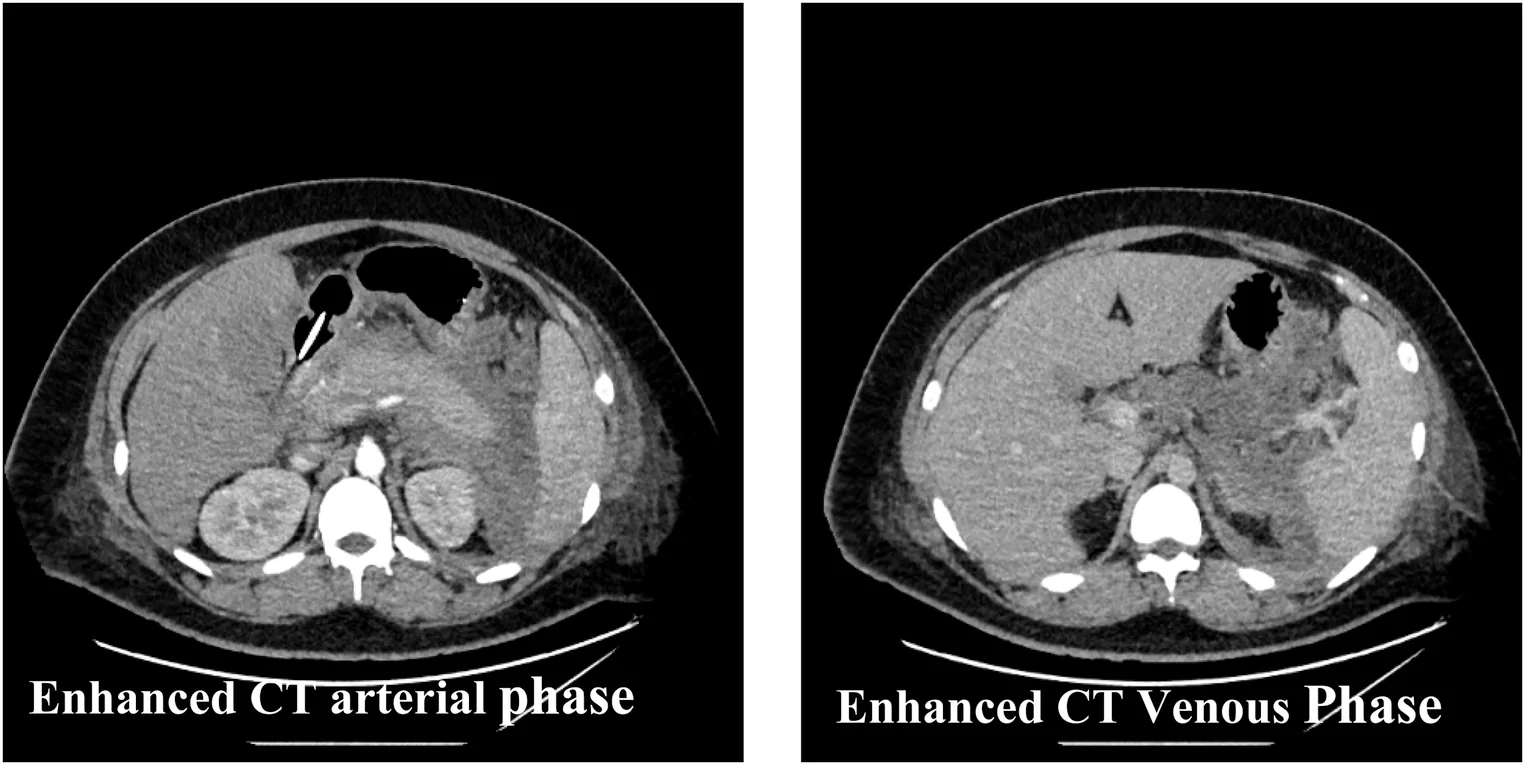
Contrast-enhanced CT during recovery: residual peripancreatic inflammation, with no pseudoaneurysm, splenic-vein thrombosis, or gastric varices.

Based on the characteristic endoscopic appearance, successful endoscopic clipping, exclusion of alternative vascular causes, and favorable clinical course, the bleeding episode was attributed primarily to rupture of a gastric Dieulafoy lesion during recovery from HTG-AP. At follow-up endoscopy approximately seven months after the bleeding episode, the three metallic clips remained *in situ*, the gastric angle ulcer had completely healed, and no recurrent bleeding was observed (Figure 6). The patient remained clinically stable without further gastrointestinal hemorrhagic events during follow-up.

**Figure 6:**
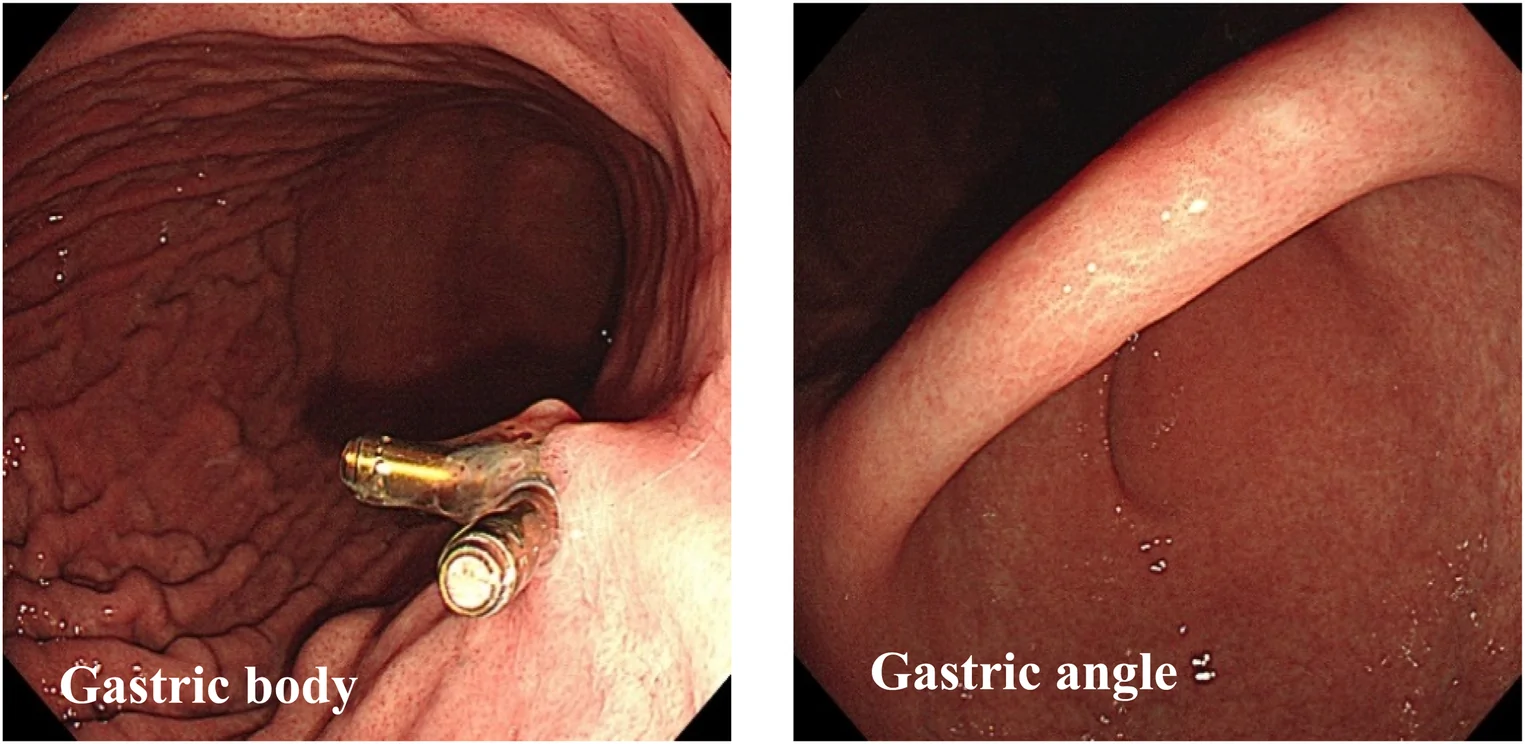
Seven-month follow-up: clips retained, mucosa healed, gastric-angle ulcer resolved, and no recurrent bleeding.

## 3 Discussion

### 3.1 Clinical significance and diagnostic challenges

The present case describes a young patient with HTG-AP who developed life-threatening upper gastrointestinal hemorrhage during the recovery phase of the disease. The bleeding source was ultimately identified as a gastric Dieulafoy lesion after an initially nondiagnostic emergency endoscopy. Successful endoscopic clipping resulted in durable hemostasis without recurrent bleeding during long-term follow-up. Several features make this case clinically noteworthy (16). First, although gastrointestinal bleeding is a recognized complication of acute pancreatitis, it is most commonly attributed to stress-related mucosal disease, peptic ulcer disease, erosive gastritis, coagulation abnormalities, or pancreatitis-associated vascular complications (17). In contrast, bleeding originating from a gastric Dieulafoy lesion in the setting of HTG-AP appears to be exceedingly uncommon (14). Second, the patient developed hemorrhage during clinical recovery rather than during the initial phase of severe systemic inflammation, potentially lowering clinical suspicion for catastrophic gastrointestinal bleeding. Third, the initial endoscopic examination failed to identify the bleeding source because of extensive intragastric clot retention, highlighting the diagnostic challenges associated with Dieulafoy lesions and the importance of repeat endoscopic evaluation (18). Taken together, this case emphasizes that uncommon vascular lesions should remain within the differential diagnosis when upper gastrointestinal bleeding occurs during recovery from acute pancreatitis, particularly when initial investigations fail to establish a definitive etiology.

One of the most instructive aspects of this case is the diagnostic difficulty encountered during the initial endoscopic evaluation. Dieulafoy lesions are notoriously challenging to diagnose because the responsible vessel is often small, bleeding may be intermittent, and the surrounding mucosa typically appears normal. In addition, active hemorrhage frequently results in extensive clot retention that obscures the lesion. Previous studies have reported that a substantial proportion of patients require more than one endoscopic examination before a definitive diagnosis is established (7, 19).

In the present patient, emergency endoscopy was performed shortly after the onset of massive hematemesis. However, the stomach contained a large volume of fresh blood and organized clots, particularly within the cardia, gastric fundus, and upper gastric body. Despite repeated irrigation and suction, visualization remained inadequate, preventing identification of the bleeding source. Importantly, persistent hemoglobin decline following the initial examination prompted repeat endoscopy after further resuscitation and partial clearance of retained clots. During the second procedure, improved visualization allowed detection of the lesion on the lesser curvature of the upper gastric body near the cardia (12).

This experience highlights a key clinical message: a negative or nondiagnostic initial endoscopy does not exclude the presence of a Dieulafoy lesion. When clinical evidence strongly suggests ongoing upper gastrointestinal bleeding, repeat endoscopy should be performed promptly after hemodynamic stabilization and optimization of visualization conditions.

### 3.2 Differential diagnosis and the role of anticoagulation

Upper gastrointestinal bleeding in patients with acute pancreatitis may result from several distinct mechanisms. Therefore, a systematic differential diagnostic approach is essential. The most common causes include stress-related mucosal injury, peptic ulcer disease, erosive gastritis, Mallory–Weiss tears, coagulation abnormalities, and anticoagulation-associated hemorrhage. In patients with severe pancreatitis, vascular complications such as splenic vein thrombosis, gastric varices, pseudoaneurysm formation, and pseudoaneurysm rupture must also be considered because these conditions may lead to massive bleeding and are associated with substantial mortality (17).

In the present case, several alternative etiologies were carefully evaluated. Follow-up endoscopy revealed a small superficial ulcer at the gastric angle and mild erosive gastritis. However, neither lesion demonstrated active bleeding nor exhibited endoscopic features consistent with recent major hemorrhage. Furthermore, the site of active bleeding was anatomically separate from these abnormalities. Pancreatitis-related vascular complications were considered because they represent a well-recognized cause of severe hemorrhage in patients with acute pancreatitis. However, repeated contrast-enhanced CT examinations did not demonstrate pseudoaneurysm formation, splenic vein thrombosis, gastric varices, or other vascular abnormalities. Therefore, these possibilities were considered unlikely. The lesion identified during repeat endoscopy demonstrated several characteristic features of Dieulafoy lesion, including an isolated protruding vessel-like structure, active oozing from an otherwise normal-appearing mucosa, absence of a surrounding ulcer crater, and immediate cessation of bleeding following mechanical clipping. Collectively, these findings strongly supported the diagnosis of gastric Dieulafoy lesion as the primary source of hemorrhage.

Another important question raised by this case concerns the contribution of anticoagulation therapy to the hemorrhagic event. The patient received low-molecular-weight heparin during hospitalization and anticoagulation associated with extracorporeal therapies. Consequently, anticoagulation-associated bleeding was initially considered. However, several observations argue against anticoagulation as the primary etiology. First, dynamic coagulation monitoring demonstrated only mild fluctuations in prothrombin time and international normalized ratio. Activated partial thromboplastin time was not significantly prolonged and was below the upper reference limit at several time points. Second, no evidence of severe coagulopathy or anticoagulation overdose was documented. Third, repeat endoscopy identified a discrete structural vascular lesion that directly explained the source of bleeding (7).

Nevertheless, anticoagulation should not be dismissed entirely. It is plausible that anticoagulant exposure increased the volume of blood loss and prolonged bleeding after rupture of the lesion. Therefore, anticoagulation may have functioned as a contributing or amplifying factor rather than the primary cause of hemorrhage. From a clinical perspective, this distinction is important because premature attribution of bleeding solely to anticoagulation may delay identification of an underlying structural lesion requiring definitive intervention (14, 20). Accordingly, anticoagulant exposure should prompt careful assessment of coagulation status and anticoagulation intensity, but it must not lead clinicians to stop searching for an organic lesion; ongoing blood loss, hemodynamic instability, or an unexplained decline in hemoglobin warrants continued evaluation for a structural bleeding source that requires definitive treatment.

### 3.3 Potential mechanisms linking HTG-AP and dieulafoy bleeding

The central interpretation is that the patient most likely harbored a pre-existing Dieulafoy lesion—a large-caliber submucosal artery coursing beneath a minute mucosal defect—that became clinically apparent and ruptured during the physiological stress of severe critical illness; this case cannot demonstrate that HTG-AP caused either the lesion or its rupture. Several factors associated with severe HTG-AP and its management may have influenced the timing or severity of the hemorrhage, although their individual roles cannot be determined from a single case: systemic inflammation, cytokine release, and endothelial-barrier or microvascular dysfunction may have increased the susceptibility of an abnormal vessel (21); coagulation–fibrinolysis activation, reflected by elevated fibrin-degradation products and D-dimer (25), may have amplified bleeding after the vessel ruptured (20, 21); plasma exchange and CRRT may have been associated with fluctuations in intravascular volume and coagulation homeostasis (22); and prolonged critical illness, enteral tube placement, and gastric decompression may have added local mechanical stress (7). None of these factors would alone create a Dieulafoy lesion. The hemorrhage was therefore likely multifactorial, arising from a pre-existing lesion against a background of inflammatory, hemodynamic, and coagulation-related disturbance, but a direct causal relationship cannot be inferred.

### 3.4 Comparison with the literature

Dieulafoy lesion accounts for approximately 1%–2% of cases of acute non-variceal upper gastrointestinal bleeding. The majority of lesions occur in the proximal stomach, particularly along the lesser curvature within several centimeters of the gastroesophageal junction. This anatomical distribution closely corresponds to the location identified in the present patient (14). Historically, surgical intervention was frequently required because diagnosis was difficult and endoscopic therapy was limited. However, advances in endoscopic techniques have dramatically improved outcomes. Current guidelines recommend endoscopic therapy as the first-line treatment for Dieulafoy lesions, with reported primary hemostasis rates exceeding 90%. Mechanical methods, including hemoclipping and endoscopic band ligation, are generally associated with lower rebleeding rates than injection therapy alone because they directly occlude the responsible vessel (7, 23). In a recent two-decade single-center series of 39 patients, one-week rebleeding and 90-day mortality rates were 33% and 28%, respectively, with lesion location, low hemoglobin, and comorbidity influencing outcomes (24). In the present case, placement of three metallic clips achieved immediate and durable hemostasis, consistent with previously reported outcomes. A review of the available literature identified only a limited number of reports describing pancreatitis-associated Dieulafoy lesions. Most involved periampullary or extra-gastric locations and were not specifically associated with hypertriglyceridemic pancreatitis (10, 12). Consequently, the present case expands the clinical spectrum of gastrointestinal bleeding encountered during HTG-AP and underscores the need for awareness of rare vascular etiologies.

### 3.5 Clinical implications and limitations

Several practical lessons can be derived from this case. First, clinicians should maintain a broad differential diagnosis when gastrointestinal bleeding occurs during recovery from acute pancreatitis. Although stress-related mucosal injury and anticoagulation-associated bleeding are common considerations, uncommon vascular lesions should not be overlooked. Second, contrast-enhanced CT is valuable for excluding pseudoaneurysm formation, splenic vein thrombosis, and other pancreatitis-related vascular complications. However, negative imaging findings do not exclude endoscopically detectable lesions such as Dieulafoy lesion. Third, repeat endoscopy should be strongly considered when the initial examination is nondiagnostic and clinical evidence of ongoing bleeding persists. Failure to identify a lesion during emergency endoscopy does not necessarily indicate its absence. Finally, in the present patient, mechanical endoscopic hemostasis using metallic clips achieved immediate and durable hemostasis; the choice of endoscopic modality should be individualized according to lesion characteristics, the patient's clinical condition, local expertise, and current clinical guidelines.

Several limitations should be acknowledged. First, this report describes a single patient, limiting the generalizability of the observations. Second, although clinical, laboratory, imaging, and endoscopic findings strongly supported the diagnosis of gastric Dieulafoy lesion, histopathological confirmation of the responsible vessel was not available. Third, the precise contribution of anticoagulation to the severity of hemorrhage could not be quantitatively assessed. Fourth, because the rarity of this clinical scenario precludes definitive causal inference, the proposed relationship between HTG-AP and Dieulafoy lesion rupture remains speculative. Fifth, the favorable response to clipping in this single patient does not establish comparative efficacy or universal safety. Finally, because of the retrospective nature of the clinical documentation, the precise organ-dysfunction criteria and treatment parameters that prompted escalation to plasma exchange and CRRT cannot be fully reconstructed; the indications reported are those available in the medical record and should not be interpreted as universal indications.

Despite these limitations, the characteristic endoscopic findings, successful mechanical hemostasis, exclusion of alternative causes, and absence of recurrent bleeding during follow-up collectively support the validity of the diagnosis and the clinical conclusions derived from this case.

## 4 Conclusion

A gastric Dieulafoy lesion is a rare but potentially life-threatening cause of upper gastrointestinal bleeding during recovery from HTG-AP. The principal lesson of this case is that a nondiagnostic initial endoscopy in the setting of extensive intragastric clot retention does not exclude this diagnosis; when bleeding persists, repeat endoscopy after hemodynamic stabilization and adequate gastric clearance is warranted, and hemorrhage should not be attributed solely to stress-related mucosal injury or anticoagulation. Although this single case cannot establish a causal relationship between HTG-AP and Dieulafoy lesion rupture or the comparative efficacy of endoscopic therapies, greater awareness of this uncommon vascular lesion may facilitate earlier diagnosis in patients recovering from complicated acute pancreatitis.

## 5 Patient perspective

The patient and his family were informed of the diagnosis, the endoscopic hemostatic procedure, the need for post-procedural monitoring, long-term lipid control, and regular follow-up after discharge. They expressed understanding that the disease had progressed rapidly and was associated with a high risk of complications. The patient and his family agreed that anonymized clinical information, laboratory data, endoscopic images, and radiological images could be used for medical publication.

## Data Availability

The original contributions presented in the study are included in the article/supplementary material, further inquiries can be directed to the corresponding author.
